# A single institution experience of the treatment of pancreatic ductal carcinoma: The demand and the role of radiation therapy

**DOI:** 10.1371/journal.pone.0227305

**Published:** 2019-12-30

**Authors:** Katsumaro Kubo, Koichi Wadasaki, Daisuke Komichi, Tamito Sasaki, Hiroyasu Yamada, Yasuhiro Matsugu, Toshiyuki Itamoto, Mihoko Doi, Katsunori Shinozaki

**Affiliations:** 1 Department of Radiation Oncology, Hiroshima High-Precision Radiotherapy Cancer Center, Hiroshima-shi, Hiroshima, Japan; 2 Department of Radiation Oncology, Hiroshima Prefectural Hospital, Hiroshima-shi, Hiroshima, Japan; 3 Department of Gastroenterology, Hiroshima Prefectural Hospital, Hiroshima-shi, Hiroshima, Japan; 4 Department of Surgery, Hiroshima Prefectural Hospital, Hiroshima-shi, Hiroshima, Japan; 5 Department of Clinical Oncology, Hiroshima Prefectural Hospital, Hiroshima-shi, Hiroshima, Japan; University of Nebraska Medical Center, UNITED STATES

## Abstract

We aimed to demonstrate a single institution experience of treatment of pancreatic ductal carcinoma and to identify the role of radiation therapy. We assessed all patients who were diagnosed with pancreatic ductal carcinoma from January 2011 to December 2017. A total of 342 patients were enrolled. Thirteen, 131, 36, and 162 patients had stage I, II, III, and IV disease, respectively (UICC TNM, 7th edition). Among the patients with stages I–III disease, 94 underwent surgery, and the median overall survival (OS) was 33 months. Of patients with stages I–III disease who were not suitable for surgery, 58 patients received chemotherapy, and the median OS was 12 months. Among them, 17 patients received chemoradiotherapy added on chemotherapy and their OS was significantly better than that of patients who received chemotherapy alone. Of patients with stage IV disease, 111 received chemotherapy, and the median OS was 6 months. This study evaluated the demand, role, and outcome of each treatment modality and demonstrated a single institution experience of treatment of pancreatic ductal carcinoma. The demand and role of radiation therapy remained small; however, radiation therapy might have some importance as a local treatment.

## Introduction

Pancreatic cancer is the fourth leading cause of cancer-related mortality in Japan. Its prognosis remains poor, and an estimated 34,200 deaths occurred in Japan in 2017 [1]. The high biologically malignant nature of pancreatic cancer facilitates infiltration into the surrounding tissues and distant metastasis. Surgical resection is a treatment with the potential for cure; however, only <20% of all patients with pancreatic cancer are diagnosed with resectable disease [2]. Therefore, for unresectable tumors that account for the majority of pancreatic cancer cases, other treatment modalities, such as radiation therapy and chemotherapy, are also important.

Various studies about the treatment depending on the status of patient’s disease (resectable, borderline resectable, unresectable tumors, and metastatic disease) have been reported. However, in Japan, no previous study has reported on the prevailing status of pancreatic cancer treatment, such as a nationwide survey. Therefore, it is difficult to understand the actual status of the treatment for this disease.

In this study, we analyzed all pancreatic cancer cases at a single institution based on their treatment, such as surgery, radiation therapy, chemotherapy, and palliative therapy. We aimed to identify the demand, role, and outcome of each treatment modality and demonstrate a single institution experience of treatment of pancreatic ductal carcinoma to understand the actual status. In particular, we focused on the demand and role of radiation therapy.

## Materials and methods

### Patients

From January 2011 to December 2017, 428 patients with pancreatic tumor were diagnosed at Hiroshima Prefectural Hospital. Among these patients, those who were pathologically or clinically diagnosed with pancreatic ductal carcinoma were included in the analysis. We did not exclude patients without a pathological diagnosis as the advancements in radiographic studies and tumor markers improved the accuracy of the clinical diagnosis, and undergoing invasive diagnostic procedures was sometimes a burden for these patients, such as those undergoing palliative treatment. We thought that the investigation including such cases was important to understand the actual state of pancreatic cancer treatment in clinical practice. Patients with pancreatic intraepithelial neoplasia, neuroendocrine carcinoma, intraductal papillary mucinous neoplasm, acinar cell carcinoma, squamous cell carcinoma, and cystadenocarcinoma were excluded. Patients whose clinical findings before treatment or the details of treatment were insufficient or those who were lost to follow-up immediately after diagnosis or treatment were also excluded.

Before therapy, all patients underwent clinical evaluation by assessment of previous medical history, physical and laboratory examinations, and radiographic studies. Most of the patients underwent endoscopic ultrasound-guided fine needle aspiration. The following laboratory examinations were performed: complete blood cell count, liver function studies, renal function studies, measurement of electrolytes, and carbohydrate antigen 19–9 assay. The following radiographic studies were performed: computed tomography of the chest, abdomen, and pelvis and magnetic resonance imaging of the liver if liver metastasis had been suspected. The clinical TNM stage was defined according to the Union for International Cancer Control tumor node metastasis classification system, 7th edition. The study protocol was approved by the Human Ethics Review Committee of Hiroshima Prefectural Hospital. The need for informed consent was waived due to the retrospective nature of this study.

### Treatment

Among patients with stages I–III disease, those with operable pancreatic cancer underwent surgery. Postoperatively, most of the patients underwent adjuvant chemotherapy. Depending on the case, postoperative radiation therapy was performed when the surgical margin was positive. Patients with borderline resectable or unresectable pancreatic cancer underwent chemotherapy. Patients in whom the tumor had shrunk after chemotherapy and had been judged to be operable underwent surgery. Chemoradiotherapy (CRT) was considered when the tumor was not suitable for surgery after several cycles of chemotherapy, but the lesions were localized. The patients who were not suitable for both surgery and chemotherapy due to their general condition or coexisting diseases received palliative therapy only. Patients with stage IV disease received chemotherapy, while those for whom chemotherapy was not suitable received palliative therapy.

### Evaluation and statistical analysis

We analyzed the overall survival (OS) rate of the entire cohort, those of patients with various stages, and that for each treatment modality. Among patients with stages I–III disease, those who underwent surgery were categorized as the surgery group, and those who received chemotherapy without surgery as the chemotherapy group. In the chemotherapy group, we compared the OS rates of patients with CRT with that of those without CRT. CRT added on chemotherapy was administered only in patients without distant metastasis after several cycles of chemotherapy (median duration, 4 months). To avoid selection bias, among patients who received chemotherapy alone, those who died or developed distant metastasis within 4 months after the first treatment were excluded from this comparison. In addition, univariate analyses using the Mantel–Haenszel χ^2^-test and multivariate analyses using logistic regression were performed to determine the statistical significance of differences in OS. Investigated factors included age, sex, performance status, tumor location, TNM stage, CA19-9 level, and presence of CRT.

The Kaplan–Meier method was used to calculate the OS rate. The OS was calculated from the date of initiation of the first treatment until the date of the final follow-up or death of any cause. The χ^2^ test or Student’s *t*-test was conducted to determine the significant differences between surgery group and chemotherapy group and between the patients who received CRT added on chemotherapy and those who received chemotherapy alone. Ekuseru-Toukei 2015 (version 1.02; Social Survey Research Information Co., Ltd., Tokyo, Japan) was used to perform the statistical analyses. Analysis items with p < 0.05 were considered statistically significant.

## Results

### Patients

Of the 428 patients who were diagnosed with pancreatic tumor at Hiroshima Prefectural Hospital, 86 were excluded from the analysis. Forty-nine patients were excluded due to their pathological diagnoses, 33 were excluded due to insufficient data on treatments, and 4 were lost to follow-up immediately after diagnosis or treatment. Therefore, 342 patients were finally enrolled in this study. The characteristics of the eligible patients are summarized in Table 1. The median age of the patients was 73 (range, 47–97) years and 51.8% of the patients were men. Two hundred and fifty-seven (75.1%) patients were diagnosed pathologically, while 85 (24.9%) were diagnosed clinically with invasive ductal carcinoma of the pancreas. One hundred and seventy-one (50.0%) patients had tumors in the head, 95 (27.8%) had tumors in the body, and 76 (22.2%) had tumors in the tail of the pancreas. Moreover, 10 (2.9%), 9 (2.6%), 215 (62.9%), and 108 (31.6%) patients developed T1, T2, T3, and T4 disease, respectively. Meanwhile, 146 (42.7%) patients had regional lymph node metastases. Thirteen (3.8%), 131 (38.3%), 36 (10.5%), and 162 (47.4%) patients had stage I, II, III, and IV disease, respectively. The median follow-up periods at the time of evaluation were 9 (range, 0–94) months in all patients and 25 (range, 5–94) months among survivors.

**Table 1:** Patient characteristics.

	Total	
	n = 342	100 (%)
Age, years, median (range)	73 (47–97)	-
Sex		
Male	177	51.8
Female	165	48.2
Pathological diagnosis		
Available	257	75.1
Not available	85	24.9
Location		
Head	171	50.0
Body	95	27.8
Tail	76	22.2
Stage		
I	13	3.8
II	131	38.3
III	36	10.5
IV	162	47.4
T stage		
T1	10	2.9
T2	9	2.6
T3	215	62.9
T4	108	31.6
N stage		
N0	196	57.3
N1	146	42.7
CA19-9, U/ml, median (range)	245 (1–921500)	-
Surgery Yes	94	27.5
RT Yes	44	12.9
(CRT added on chemotherapy)	17	
(Pre/postoperative RT)	5	
(RT for recurrence or metastasis)	22	
Chemotherapy Yes	251	73.4
(Neoadjuvant/adjuvant)	82	
(Without surgery)	169	
Palliative therapy alone Yes	79	23.1

RT, radiation therapy; CRT, chemoradiotherapy; CA19-9, carbohydrate antigen 19–9

Ninety-four (27.5%) patients underwent surgery. Among them, 82 patients received neoadjuvant and/or adjuvant chemotherapy. Meanwhile, 251 (73.4%) patients underwent chemotherapy. Except for those who received palliative therapy only, 95.4% of patients received chemotherapy. Forty-four (12.9%) patients received radiation therapy. Among them, pre/postoperative radiation therapy was administered to 5 patients, CRT added on chemotherapy for local control was administered to 17 patients, and radiation therapy for recurrence or metastasis was administered to 22 patients. Seventy-nine (23.1%) patients received palliative therapy only.

### Outcomes of all patients

**Fig 1:**
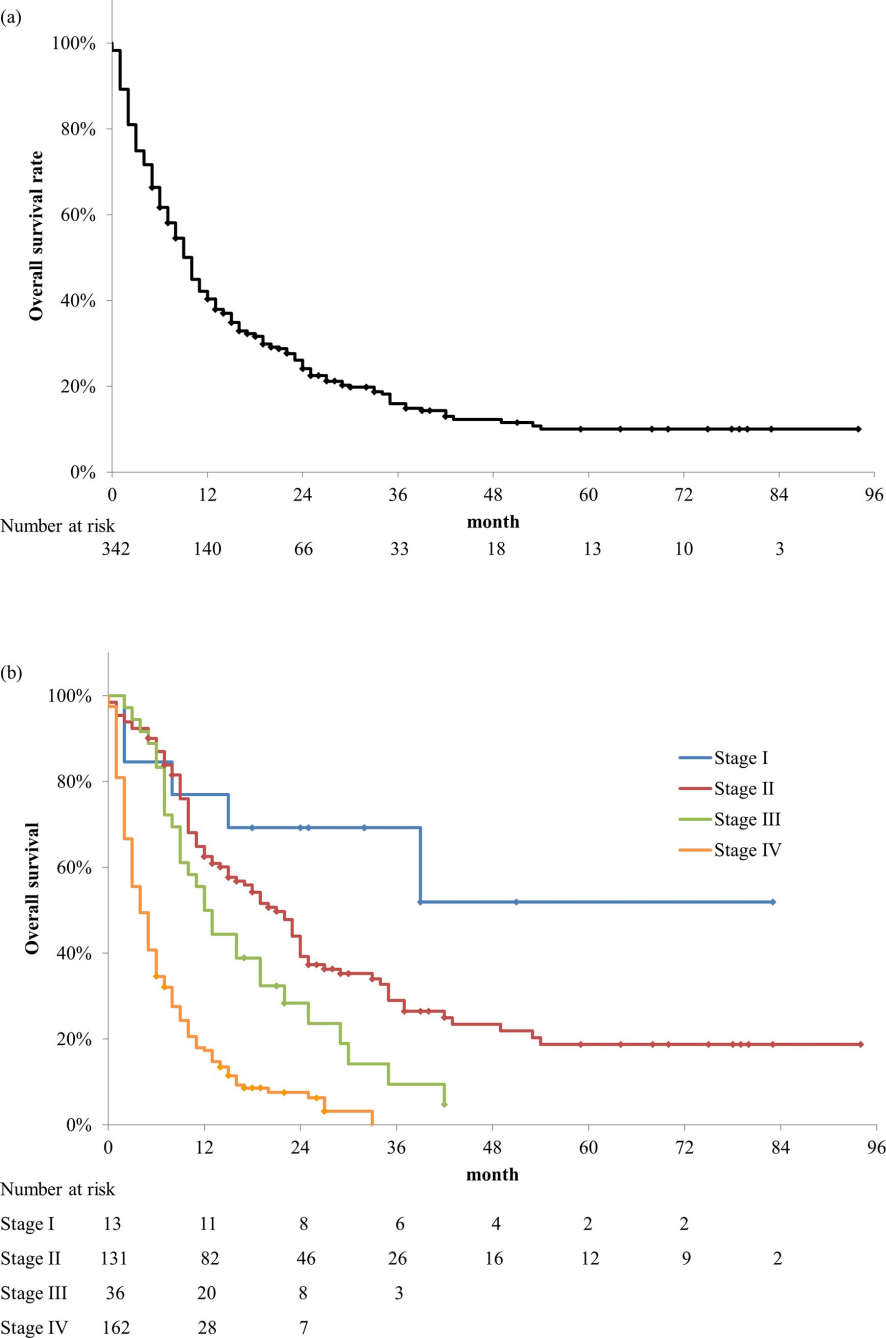
Overall survival (OS) rate of the entire patient cohort and the individual OS rates by stage. (a) OS rate of the entire patient cohort. The median OS of all patients was 9 months. The 3-year OS rate was 16.0%. (b) OS rates by stage. The 3-year OS rates of patients with stage I, II, III, and IV disease were 69.2%, 29.0%, 9.5%, and 0%, respectively.

Fig 1 shows the OS rates of the entire patient cohort and those with various stages of pancreatic cancer. The median OS of all patients was 9 (95% confidence interval [CI], 7.8–10.2) months. The 3-year OS rate was 16.0% (95% CI, 11.5–20.5). The 3-year OS rates of patients with stage I, II, III, and IV disease were 69.2% (95% CI, 44.1–94.3), 29.0% (95% CI, 20.2–37.8), 9.5% (95% CI, 0–21.3), and 0% (95% CI, 0–0), respectively. During the follow-up period, 276 (80.7%) patients died; among these, 273 died of pancreatic cancer and only 3 died of other diseases.

### Stages I–III pancreatic cancer

#### Surgery group

**Fig 2:**
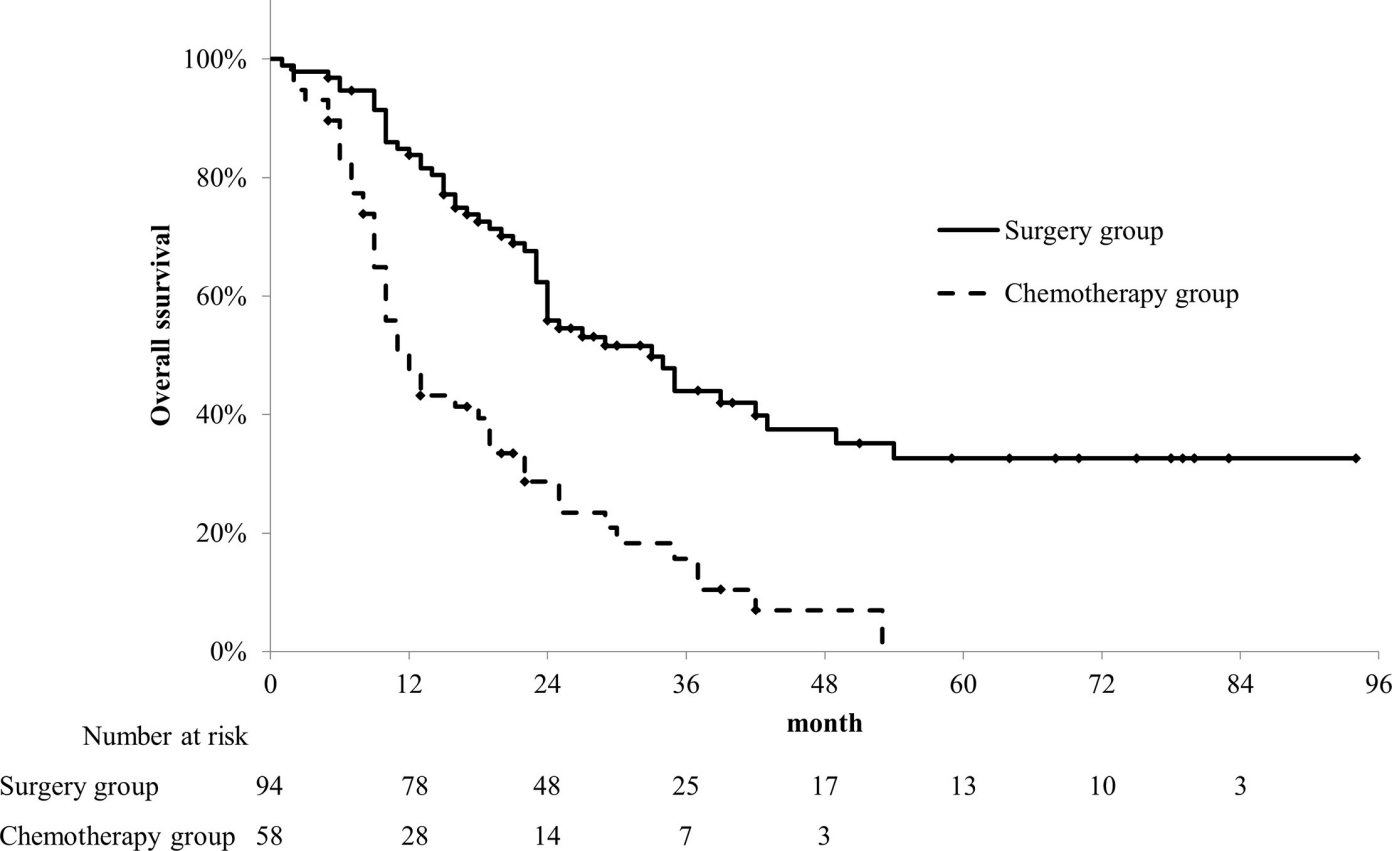
Overall survival (OS) rates of the surgery and chemotherapy groups. The median OS periods of the surgery and chemotherapy groups were 33 and 12 months, respectively. The 3-year OS rates of the surgery and chemotherapy groups were 44.0% and 15.7%, respectively.

The characteristics of the surgery group are summarized in Table 2. The median age was 73 (range, 47–88) years. Among them, 9 (9.6%), 83 (88.3%), and 2 (2.1%) patients had stages I, II, and III disease, respectively. Sixty-two patients underwent pancreatoduodenectomy, while 32 underwent distal pancreatectomy. Neoadjuvant chemotherapy was administered to 6 patients, and adjuvant chemotherapy to 80 patients. Among them, 5 patients underwent pre- or postoperative radiation therapy. The median OS period was 33 (95% CI, 22.3–43.7) months, and the 3-year OS rate was 44.0% (95% CI, 32.5–55.6) (Fig 2). In the surgery group, 44 (46.8%) patients were still alive at the time of evaluation and 12 (12.8%) survived for more than 5 years.

**Table 2:** Characteristics of patients from the surgery and chemotherapy groups.

	Surgery group		Chemotherapy group		*P* value
	n = 94	100 (%)	n = 58	100 (%)	
Age, years, median (range)	73 (47–88)	-	71 (47–91)	-	0.3963
Sex					0.5559
Male	50	53.2	28	48.3	
Female	44	46.8	30	51.7	
Pathological diagnosis					<0.0001
Available	94	100	47	81.0	
Not available	0	0	11	19.0	
Location					0.4720
Head	59	62.8	33	56.9	
Body	26	27.7	21	36.2	
Tail	9	9.6	4	6.9	
Stage					<0.0001
I	9	9.6	1	1.7	
II	83	88.3	29	50.0	
III	2	2.1	28	48.3	
T stage					<0.0001
T1	7	7.4	1	1.7	
T2	4	4.3	0	0	
T3	81	86.2	29	50.0	
T4	2	2.1	28	48.3	
N stage					0.2068
N0	72	76.6	39	67.2	
N1	22	23.4	19	32.8	
CA19-9, U/ml, median (range)	95.5 (1–7454)	-	219.5 (1–68811)	-	0.0988

CA19-9, carbohydrate antigen 19–9

#### Chemotherapy group

Among patients with stages I–III disease, 86 were not suitable for surgery. Among them, 28 patients received palliative therapy only, and the other 58 patients received chemotherapy. The characteristics of the chemotherapy group are summarized in Table 2. The median age of the chemotherapy group was 71 (range, 47–97) years. Among them, 1 (1.7%), 29 (50.0%), and 28 (48.3%) patients had stages I, II, and III disease, respectively. The medications administered as initial treatment were gemcitabine in 23 patients, gemcitabine plus S-1 in 5 patients, gemcitabine plus nanoparticle albumin-bound (nab)-paclitaxel in 9 patients, FOLFIRINOX (combination chemotherapy regimen consisting of oxaliplatin, irinotecan, fluorouracil, and leucovorin) in 13 patients, and others in 8 patients, respectively. In the chemotherapy group, the median OS was 12 months (95% CI, 8.9–15.1), and the 3-year OS rate was 15.7% (95% CI, 4.8–26.5) (Fig 2).

**Fig 3:**
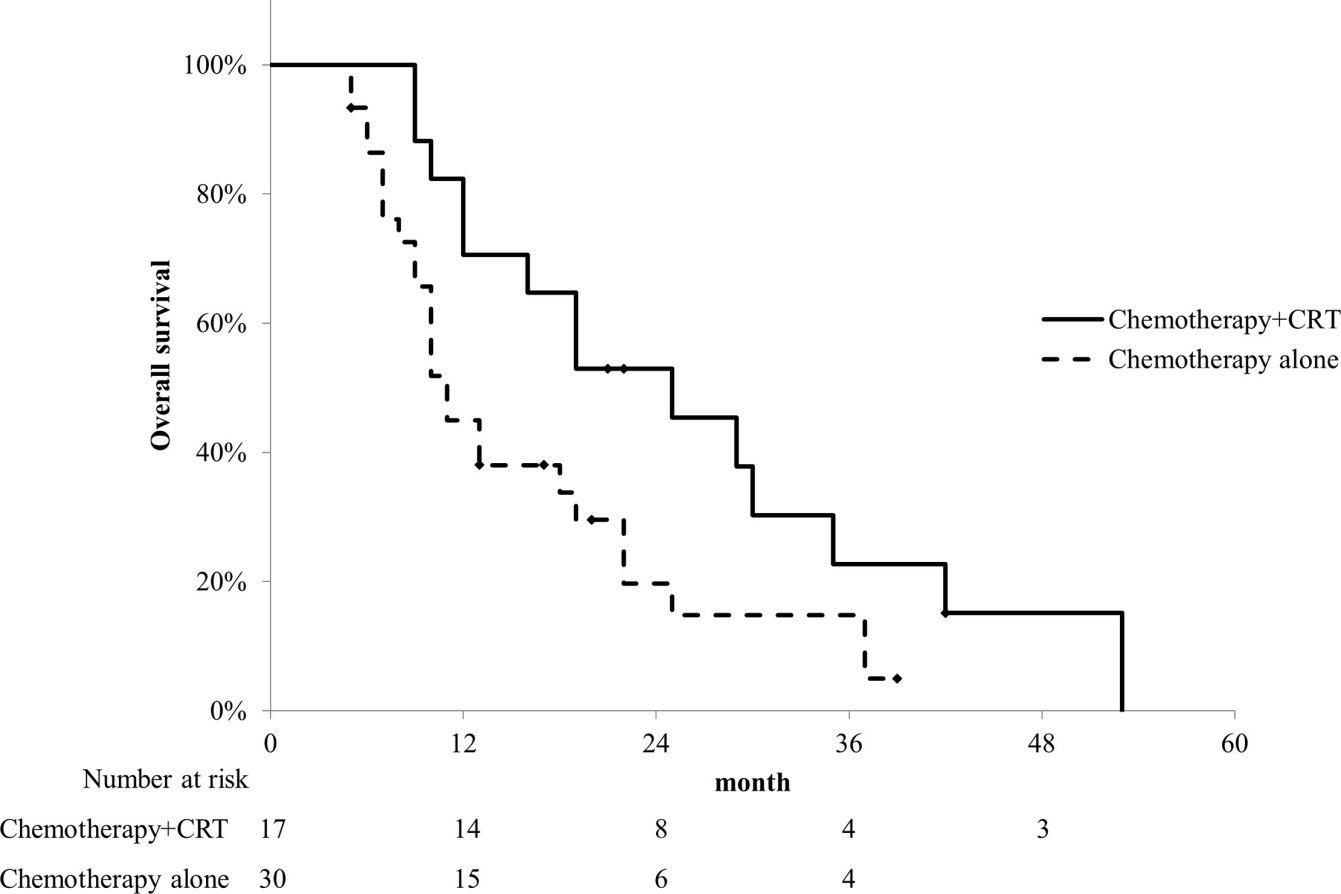
Comparison of overall survival (OS) between chemoradiotherapy added on chemotherapy and chemotherapy alone in the chemotherapy group. The median OS of the patients who received chemoradiotherapy added on chemotherapy was significantly better than that of patients who received chemotherapy alone (25 vs 10 months, p = 0.0320).

In the chemotherapy group, 17 patients received CRT added on chemotherapy. Three-dimensional conformal radiation therapy was used for treatment planning. Primary tumors and lymph node metastases with adequate margins were irradiated. The total prescribed dose was 50.4 Gy using 6–10 MV photon beams (1.8 Gy/fraction). Only 3 patients received proton beam therapy. The prescribed dose was 67.5 GyE (gray equivalent) using proton beams (2.7 GyE/fraction). The median duration from initial chemotherapy to CRT was 4 (range, 0–11) months. All patients received concurrent chemotherapy. S-1 was administered to 11 patients and gemcitabine to 6 patients. Fig 3 shows a comparison of the OS between the patients who received CRT added on chemotherapy and those who received chemotherapy alone. No significant differences in patient or tumor characteristics were observed between groups, except in terms of age (Table 3). The median OS of the patients who received CRT added on chemotherapy was significantly better than that of those who received chemotherapy alone (25 vs 11 months, p = 0.0320). Table 4 shows the univariate and multivariate analyses of OS. Performance status (0 or 1) and CRT added on chemotherapy were significant prognostic factors of OS in the univariate analysis and performance status (0 or 1) remained significant in the multivariate analysis (p = 0.0125). The patients who received CRT added on chemotherapy tended to have good prognoses; however, CRT added on chemotherapy was not significant in the multivariate analysis (p = 0.0751).

**Table 3:** Comparison of chemoradiotherapy added on chemotherapy and chemotherapy alone in the chemotherapy group.

	Chemotherapy + CRT		Chemotherapy alone		*P* value
	n = 17	100 (%)	n = 30	100 (%)	
Age, years, median (range)	64 (54–80)	-	76 (47–91)	-	0.0002
Sex					1.0000
Male	9	52.9	16	53.3	
Female	8	47.1	14	47.7	
Performance status					0.2871
0–1	16	94.1	25	83.3	
2	1	5.9	5	16.7	
Pathological diagnosis					0.1163
Available	16	94.1	21	70.0	
Not available	1	5.9	9	30.0	
Location					0.8335
Head	9	52.9	17	56.7	
Body	6	35.3	11	36.7	
Tail	2	11.8	2	6.6	
Stage					0.0869
I	0	0	0	0	
II	5	29.4	18	60.0	
III	12	70.6	12	40.0	
T stage					0.0869
T3	5	29.4	18	60.0	
T4	12	70.6	12	40.0	
N stage					1.0000
N0	11	64.7	20	66.7	
N1	6	35.3	10	33.3	
CA19-9, U/ml, median (range)	208 (1–3813)	-	174 (1–9754)	-	0.8632

Among patients who received chemotherapy alone, those who died or developed distant metastasis 4 months after the first treatment were excluded from this comparison.

CRT, chemoradiotherapy; CA19-9, carbohydrate antigen 19–9

**Table 4:** Prognostic factors in the chemotherapy group; univariate and multivariate analysis.

		Median OS	UVA	MVA	Hazard ratio
		(month)	*P* value	*P* value	(95% CI)
Age, years	< 70	19	0.0582	-	-
	≥ 70	11			
Sex	Male	18	0.3395	-	-
	Female	13			
Performance status	0 or 1	19	0.0019	0.0125	0.28 (0.11–0.76)
	2	9			
Location	Head	12	0.9435	-	-
	Body or Tail	18			
Stage	II	12	0.9815	-	-
	III	19			
T stage	3	12	0.9815	-	-
	4	19			
N stage	0	19	0.1540	-	-
	1	11			
CA19-9, U/ml	< 250	19	0.2020	-	-
	≥ 250	12			
Presence of CRT	Yes	25	0.0320	0.0751	0.53 (0.26–1.07)
	No	11			

Among patients who received chemotherapy alone, those who died or developed distant metastasis 4 months after the first treatment were excluded from these analyses.

OS, overall survival; UVA, univariate analysis; MVA, multivariate analysis; CI, confidence interval; CA19-9, carbohydrate antigen 19–9; CRT, chemoradiotherapy

### Stage IV pancreatic cancer

**Fig 4:**
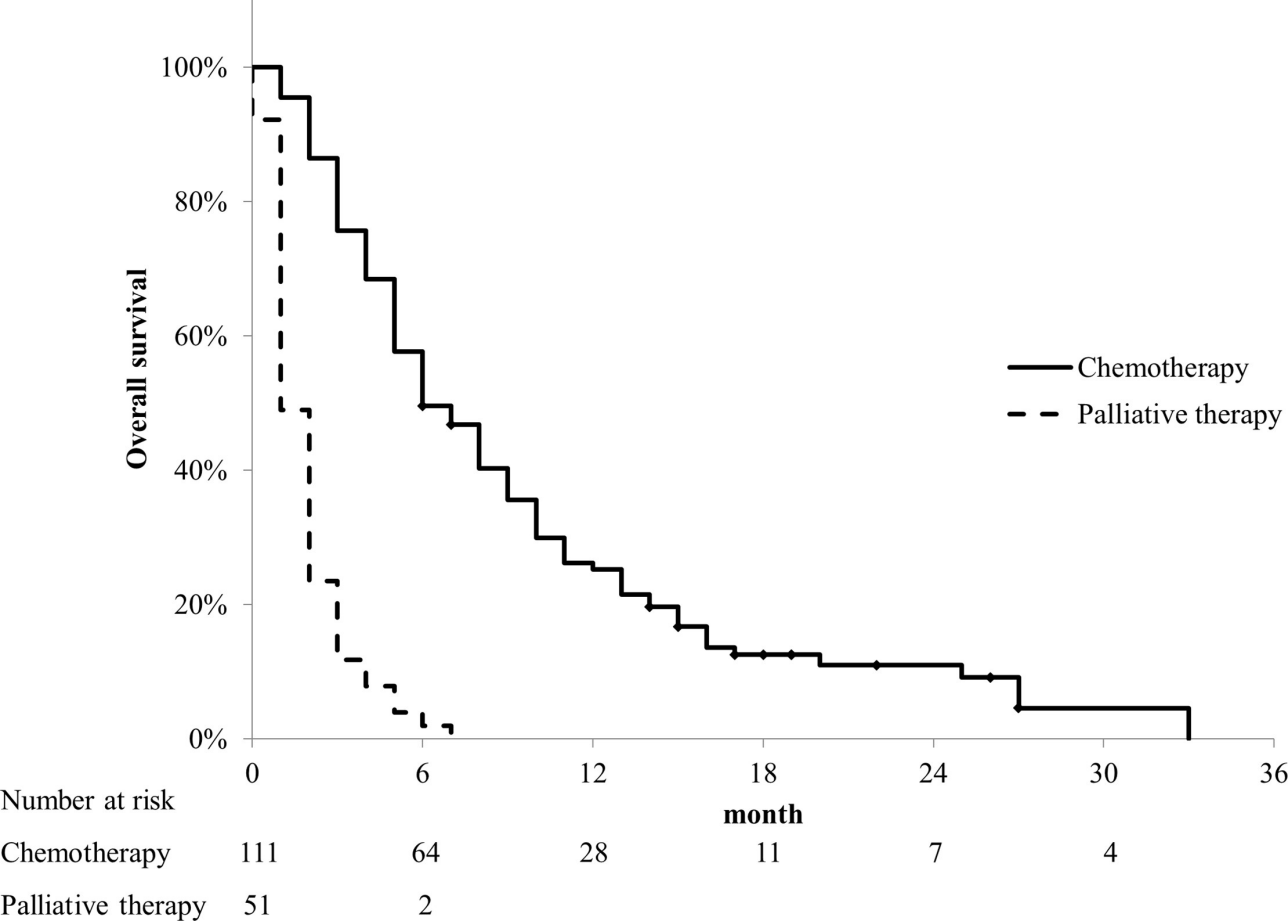
Overall survival (OS) of patients with stage IV disease who received chemotherapy and those who received palliative therapy. The median OS periods of patients with stage IV disease who received chemotherapy and those who received palliative therapy were 6 months and 1 month, respectively.

Of the 162 patients who had stage IV disease, 111 received chemotherapy. The medications administered as initial treatment were gemcitabine in 61 patients, gemcitabine plus S-1 in 9 patients, gemcitabine plus nab-paclitaxel in 17 patients, FOLFIRINOX in 13 patients, and others in 11 patients, respectively. Fifty-one patients received palliative therapy only. Fig 4 shows the OS of the patients with stage IV disease who received chemotherapy and those who received palliative therapy only. The median OS periods of the patients who received chemotherapy and those who received palliative therapy only were 6 months (95% CI, 4.4–7.6) and 1 month (95% CI, 0.7–1.3), respectively.

## Discussion

Pancreatic cancer is considered a life-threatening disease and has a high distant metastasis rate of 40–45% at diagnosis [3]. About half of the patients in this study had metastatic disease. Only 3.8% of patients had stage I disease, 38.3% had stage II disease, and 10.5% had stage III disease. About half of patients with stages I–III disease underwent surgery, 30% received chemotherapy without surgery, and 20% received palliative therapy only. In the chemotherapy group, 30% of the patients received CRT added on chemotherapy. Approximately 70% of patients with stage IV disease received chemotherapy, and 30% received palliative therapy only. Of the entire patient cohort, about 30% underwent surgery, about half received chemotherapy without surgery, and 20% received palliative therapy only. Only 5% of the entire patient cohort received CRT added on chemotherapy for local control.

Although surgery is generally a small part of the entire pancreatic cancer treatment, the proportion of patients who underwent surgery in this study was relatively high compared with that reported in other studies [2, 3]. The advancements in diagnostic studies and the current status of new chemotherapy regimens might have contributed to this result. In addition, at our institution, we discuss the treatment of each pancreatic cancer patient on the cancer board, and the relatively high number of patients who underwent surgery might be the result of appropriate case assignment. The outcomes of patients who underwent surgery were relatively good. The 3-year OS rate was 44.0%, and 12 patients survived for more than 5 years. Certainly, most patients in the surgery group had stage I or II disease, and their general conditions were relatively good, but surgery was a potentially curative treatment for those patients.

The demand for chemotherapy in the treatment of all stages of pancreatic cancer was extremely high. In this study, 95.4% of patients received chemotherapy, except for those who received palliative therapy only. The role of chemotherapy is important in various situations: in patients with metastatic disease, in patients who require adjuvant therapy, and in patients who require a combination of radiation and chemotherapy. However, the treatment outcome of patients with stages I–III disease who received chemotherapy without surgery was poorer than that of patients who underwent surgery, and chemotherapy was not a curative treatment. Of course, it had to be taken into consideration that the chemotherapy group had more advanced cases. In addition, combination chemotherapy regimens, such as FOLFIRINOX and nab-paclitaxel plus gemcitabine, were shown to be effective for metastatic pancreatic cancer and were recommended in Japan in 2016 [4–7]; however, in this study, the proportion of new regimens was small due to their novelty. Therefore, the use of these regimens is expected to increase and the treatment outcome of chemotherapy for pancreatic cancer will further improve in the future.

In patients with stages I–III disease, who were not suitable for surgery, the demand of CRT for local control was small. In addition, CRT added on chemotherapy was significant in the univariate analysis, but was not significant in the multivariate analysis. However, we presume that CRT added on chemotherapy had some advantages. The reason was that the OS of the patients who received CRT added on chemotherapy was better than that of those who received chemotherapy alone. We excluded the patients who immediately died or developed metastatic disease after receiving the first few cycles of systemic chemotherapy from this comparison to avoid selection bias. In addition, there were no significant differences between patient and tumor factors in the two groups, except for age. CRT added on chemotherapy was not significant in the multivariate analysis, but the patients who received CRT added on chemotherapy tended to have a good prognosis, and we thought there would have been some positive effect. On the other hand, radiation therapy could not be considered as curative treatment as it was difficult to administer a high radiation dose in three-dimensional conformal radiation therapy due to the anatomical location of the pancreas, and none of the patients who received CRT survived for more than 5 years in this study. The effectiveness of CRT following chemotherapy had been reported in several studies, but treatment results were not satisfactory and the median OS ranged from 11.9 to 18.7 months [8–14]. Recently, several studies reported the new radiation techniques for pancreatic cancer, such as intensity-modulated radiation therapy, stereotactic body radiation therapy, and particle radiation therapy [15–19]. In the future, these techniques might be considered as curative treatments.

In addition, we refer to radiation therapy as a part of the multidisciplinary treatment. Among the patients treated with radiation therapy in this study, 5 who underwent radiation therapy before or after surgery survived for a long time (median, 79 months). The subsets of patients, such as those with positive surgical margins, may be more likely to benefit from adjuvant CRT [8, 20]. The effectiveness of neoadjuvant CRT for borderline resectable pancreatic cancer has been reported in several studies. However, certain factors remained controversial, such as eligible criteria and results of the comparison between neoadjuvant CRT and neoadjuvant chemotherapy [21–23]. In this study, neoadjuvant CRT was given in only one patient whose tumor decreased in size after CRT and became resectable. These approaches required enough discussion; besides, the role of radiation therapy as a part of the multidisciplinary treatment seemed to be important.

This study reported on the demand, role, and outcome of each treatment modality for pancreatic cancer in a single institution. Although it was retrospective in nature and used a small sample size, this study was meaningful in reporting pancreatic cancer treatment. The role of surgery as potentially curative treatment and the high demand of chemotherapy as treatment for pancreatic cancer were confirmed in this study. The demand and role of radiation therapy in the treatment of all stages of pancreatic cancer remained small in actual clinical practice; however, radiation therapy might have some importance as a local treatment depending on the status of the patient.
